# Harmful Algal Bloom Characterization at Ultra-High Spatial and Temporal Resolution Using Small Unmanned Aircraft Systems

**DOI:** 10.3390/toxins7041065

**Published:** 2015-03-27

**Authors:** Deon Van der Merwe, Kevin P. Price

**Affiliations:** 1 Department of Diagnostic Medicine/Pathobiology, College of Veterinary Medicine, Kansas State University, 1800 Denison Ave, Manhattan, KS 66506, USA; 2 Department of Agronomy, College of Agriculture, Kansas State University, 2004 Throckmorton Plant Science Center, Manhattan, KS 66506, USA; E-Mail: kpprice@ksu.edu

**Keywords:** HAB, sUAS, cyanotoxins, cyanobacteria, blue-green algae, remote sensing

## Abstract

Harmful algal blooms (HABs) degrade water quality and produce toxins. The spatial distribution of HAbs may change rapidly due to variations wind, water currents, and population dynamics. Risk assessments, based on traditional sampling methods, are hampered by the sparseness of water sample data points, and delays between sampling and the availability of results. There is a need for local risk assessment and risk management at the spatial and temporal resolution relevant to local human and animal interactions at specific sites and times. Small, unmanned aircraft systems can gather color-infrared reflectance data at appropriate spatial and temporal resolutions, with full control over data collection timing, and short intervals between data gathering and result availability. Data can be interpreted qualitatively, or by generating a blue normalized difference vegetation index (BNDVI) that is correlated with cyanobacterial biomass densities at the water surface, as estimated using a buoyant packed cell volume (BPCV). Correlations between BNDVI and BPCV follow a logarithmic model, with *r*^2^-values under field conditions from 0.77 to 0.87. These methods provide valuable information that is complimentary to risk assessment data derived from traditional risk assessment methods, and could help to improve risk management at the local level.

## 1. Introduction

Cyanobacteria are photosynthesizing prokaryotic bacteria. They are found in most surface waters worldwide, and are often important primary producers in aquatic ecosystems. Under certain environmental conditions, however, they may proliferate exponentially to form harmful algal blooms (HABs). The incidence rates of HABs have increased over time, and higher incidence rates have been linked to excess nutrient influx into surface waters [1,2,3]. The relentless and necessary pursuit of ever-higher agricultural production to feed growing human and livestock populations inevitably results in nutrient-rich agricultural run-off, and the trend towards more frequent HABs in surface freshwaters is therefore expected to persist unless alternative, less polluting food production methods are found and implemented.

Several common genera of cyanobacteria have the ability to produce toxins, referred to as cyanotoxins, which affect people, livestock, pets, and wildlife [4,5]. The production of toxins is influenced by algal density, genetic potential, and environmental factors. Important environmental factors include nutrient concentrations, water temperature, light intensity, water pH, wind conditions, and interactions between aquatic organisms, such as predation and competition for nutrients. Toxin production is generally more common during warmer weather in summer, but can occur at any time of the year [6,7,8,9]. Animals may be exposed to cyanotoxins when drinking from, wading in, or swimming in contaminated lakes and ponds. People are most often exposed to cyanotoxins when swimming, skiing, or boating in contaminated waters. Other routes of human exposure include drinking water, contaminated foods, and nutritional supplements [10].

Traditional risk assessments depend on the interpretation of chlorophyll-a concentrations, cell density, and toxin concentration data obtained from surface water samples, usually taken at points along a lake shoreline or from a boat [11]. Results from such samples are typically available from a laboratory after a delay of a few days. The spatial and temporal sparseness of water samples taken and analyzed in the traditional manner create challenges for adequate and timely risk assessment. HABs are highly variable over space and time, and high risk of poisoning may therefore exist where traditional methods of risk assessment indicated low risk. For example, a water sample collected at the site of lethal poisoning of a dog at Milford Lake in Kansas, USA, in 2011, within one day of the exposure, indicated a safe density of *Microcystis* cells, and a safe concentration of cyanotoxins [12]. There is, therefore, a need for a more immediate, efficient, and locally relevant method of risk assessment to enable effective and appropriate local risk management. Remote sensing offers an alternative to direct water sampling for determining the presence of HABs, and can be a valuable supplement to direct water sampling in the process of risk assessment. It has been used to detect and quantify HABs based on visual identification of scums, and estimates of phycocyanin and chlorophyll-a concentrations [13,14,15,16,17].

The purpose of this study was to assess the use of small, unmanned aircraft systems (sUAS), and cameras modified to capture near infrared (NIR) and blue light wavelengths to produce color-infrared reflectance data, for remote sensing of cyanobacteria density in surface freshwaters at spatial and temporal resolutions needed for effective local risk assessment.

## 2. Results and Discussion

### 2.1. Results

#### 2.1.1. HAB Density Variation over Space and Time

**Figure 1:**
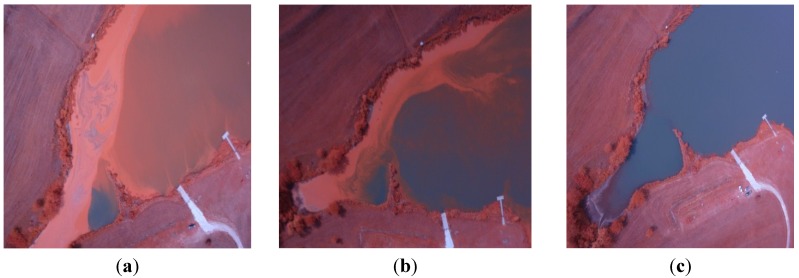
Color-infrared images derived from a small, unmanned aircraft system of a *Microcystis* HAB in Centralia Lake, KS, USA in 2012, on (**a**) 31 August; (**b**) 14 September; and (**c**) 24 September.

Repeated flights during a *Microcystis* HAB over Centralia Lake, KS, on 31 August, 14 September, and 24 September of 2012 followed by qualitative assessment of the resulting aerial images, revealed a highly complex distribution of cyanobacterial biomass at the water surface over space and time. Shorelines on different sides of the cove, where accumulation occurred, showed different cyanobacterial biomass densities on different shoreline directions, and also over short distances along the shoreline. Marked changes occurred over time (Figure 1).

#### 2.1.2. Buoyant Packed Cell Volume (BPCV) to Blue Normalized Difference Vegetation Index (BNDVI) Correlation

**Figure 2:**
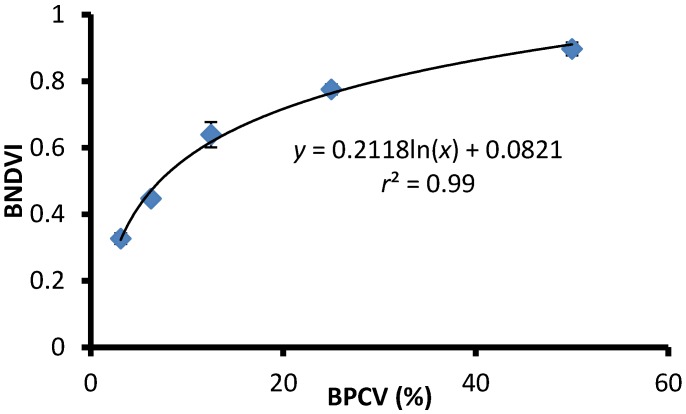
The correlation between Blue Normalized Difference Vegetation Index (BNDVI) and Buoyant Packed Cell Volume (BPCV) derived from a serially diluted sample of a single species of cyanobacteria in the genus *Microcystis*.

**Figure 3:**
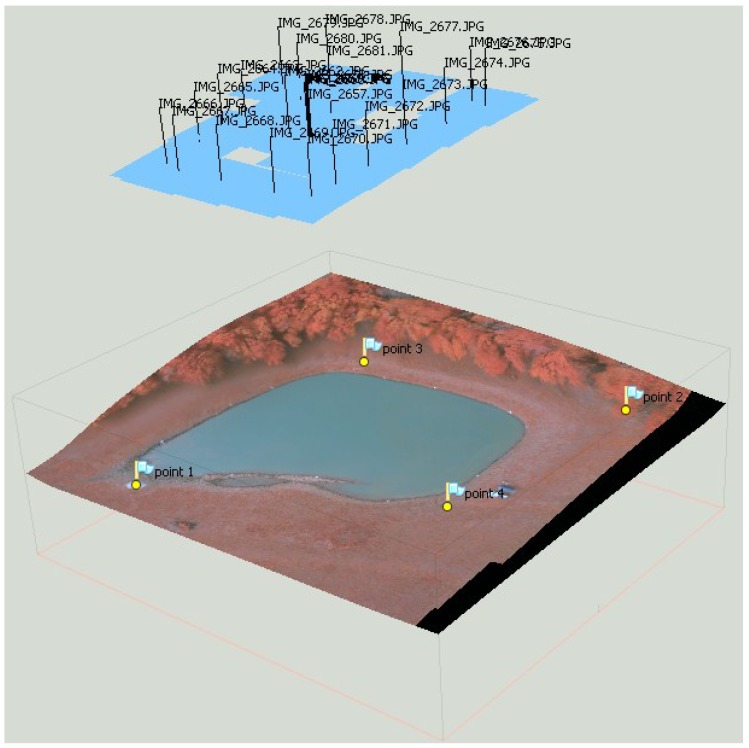
A texturized surface model derived from a livestock drinking water pond containing a harmful *Microcystis* algal bloom, including the calculated positions and orientations of images used to produce the surface model.

**Figure 4:**
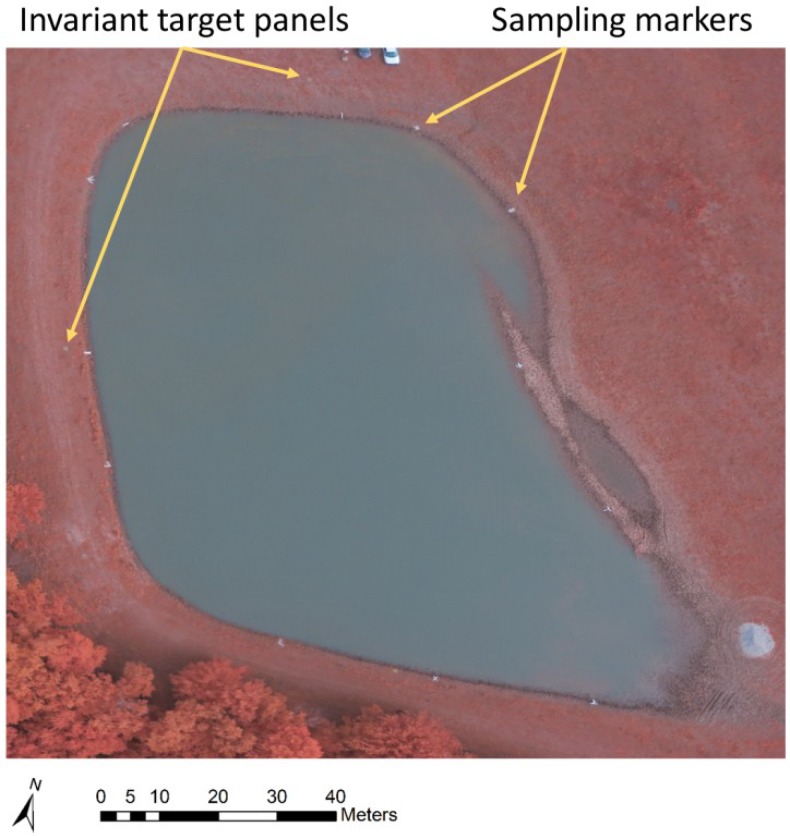
An averaged color-infrared orthomosaic of a livestock drinking water pond, derived from 25 aerial images captured at an altitude of 25 m, between 10:30 a.m. and 11:00 a.m. on 23 August, 2013. Average reflectance values for each point on the water surface were derived from 15–25 images.

**Figure 5:**
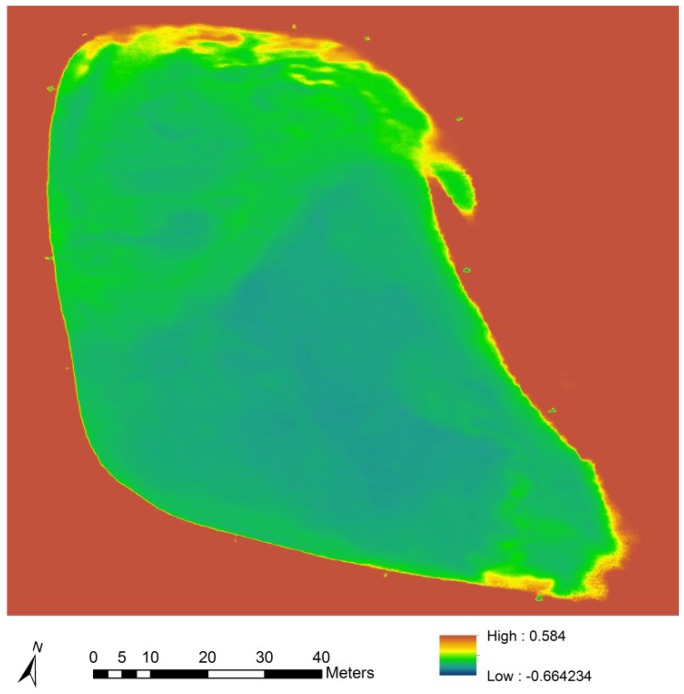
A colorized gradient map of blue normalized difference vegetation index values of a livestock drinking water pond, derived from averaged reflectance values from 25 color-infrared aerial images captured at an altitude of 50 m.

BPCV correlated strongly with blue NDVI when assessing serially diluted samples under laboratory conditions, with an *r^2^*-value of 0.99, following a logarithmic model because blue NDVI tends to become saturated towards high BPCV levels (Figure 2, Figure 3, Figure 4 and Figure 5):

(1) Blue NDVI = 0.2118ln(BPCV) + 0.0821

**Figure 6:**
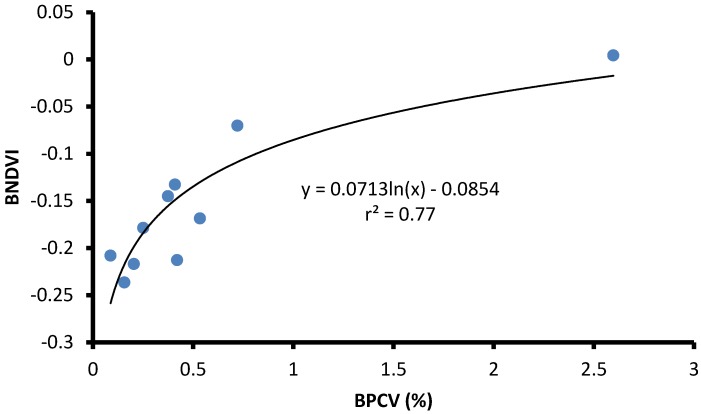
The correlation between Blue Normalized Difference Vegetation Index (BNDVI) and Buoyant Packed Cell Volume (BPCV) at a farm pond containing a harmful *Microcystis* algal bloom.

**Figure 7:**
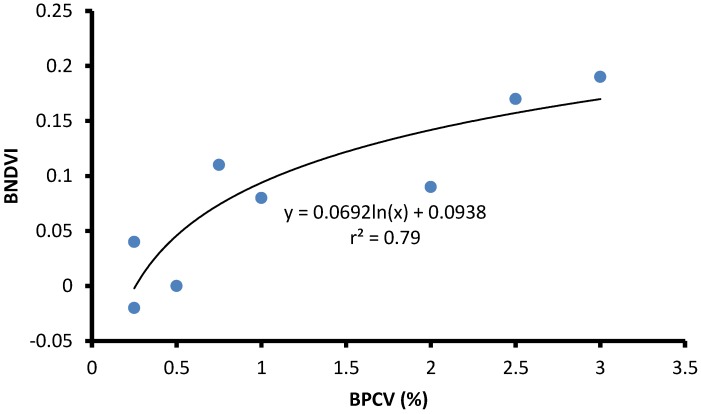
The correlation between Blue Normalized Difference Vegetation Index (BNDVI) and Buoyant Packed Cell Volume (BPCV) at a farm pond containing a harmful *Microcystis* algal bloom.

**Figure 8:**
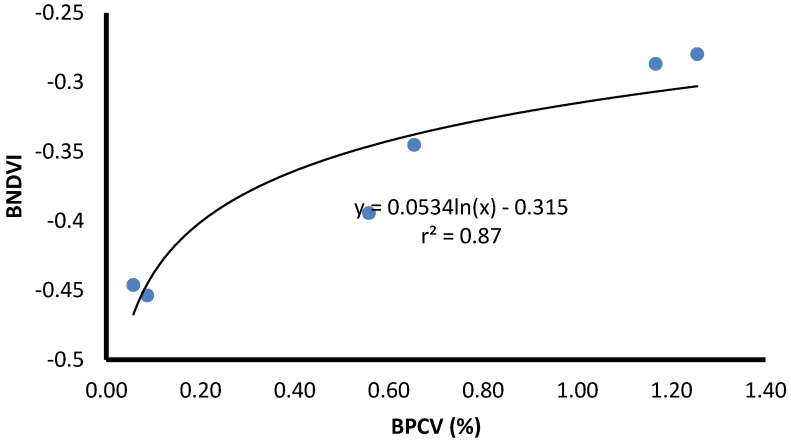
The correlation between Blue Normalized Difference Vegetation Index (BNDVI) and Buoyant Packed Cell Volume (BPCV) at a farm pond containing a harmful *Microcystis* algal bloom.

Three ponds were investigated to determine BPCV to blue NDVI correlations under field conditions. Logarithmic models provided good correlations for two ponds with pure *Microcystis* blooms (Equations (2) and (3); Figure 6 and Figure 7), and a pond with a mixed *Microcystis* and *Aphanizomenon* bloom (Equation (4); Figure 8). Correlation between BPCV and blue NDVI remained high under field conditions, with *r^2^*-values based on logarithmic models of 0.77, 0.79, and 0.87, respectively, for Pond 1, Pond 2, and Pond 3. The model parameters were as follows:

(2) BNDVI = 0.0713ln(BPCV) – 0.0854

(3) BNDVI = 0.0692ln(BPCV) + 0.0938

(4) BNDVI = 0.0534ln(BPCV) – 0.315

Graphic depictions of results from pond 3 are represented in Figure 3, Figure 4, Figure 5 and Figure 8.

### 2.2. Discussion

The use of satellite imagery for assessment of cyanobacteria cell densities in surface waters has been well established, both for early detection and monitoring purposes, and is the preferred method for tracking blooms in oceans and large lakes [18,19,20,21]. Manned aircraft are typically able to able to carry larger and heavier payloads compared to sUAS, can stay airborne for longer, and can operate at higher altitudes. It allows manned aircraft to deploy relatively large, hyperspectral sensors that are able to differentiate relatively narrow spectral bands, over relatively large areas. The use of narrow spectral bands can be an important advantage in differentiating cyanobacteria from other photosynthetic organisms based on unique reflectance characteristics associated with pigments produced by cyanobacteria [18]. The ability to differentiate cyanobacteria from other photosynthetic organisms can be a major advantage in situations where direct water sampling and access to *in situ* data are not available. The ability of manned aircraft to cover larger areas compared to sUAS, also makes them suited for assessment of lakes that are too large for sUAS to cover effectively. It is therefore important to assess the potential role of sUAS in remote sensing in relation to traditional remote sensing approaches. Although satellite and manned aircraft remote sensing are extremely useful and are expected to continue to be important, they are limited for use in rapid, local risk assessment, particularly where there is a need for water quality information on small aquatic environments or specific areas along lake shorelines, by the cost and size of narrow band multispectral and hyperspectral sensors fitted with custom-made optics, the cost of manned aircraft and satellite operations, observation limitations imposed by cloud cover, the frequency of suitable satellite positioning over the target area, and spatial resolutions that are often inadequate for small water bodies such as livestock ponds and the detection of small pockets of cyanobacterial biomass accumulation. The different types of remote sensing therefore do not compete directly with each other, but rather provide complimentary information to the risk assessment process.

The spatial distribution of cyanobacteria over space and time is highly variable and complex, to the extent that sampling at a specific location may only be relevant to risk at that location around the time of sampling. Pockets of high biomass accumulations may be intermixed, over short distances, with areas of low biomass accumulation. Average cyanobacterial density in a lake or pond is therefore only partially related to local risk, and a large number of samples are required to produce a statistically robust estimate. These factors make it difficult to predict the local risk along a shoreline, or at specific points in a lake, based on averages derived from sparse data points generated by traditional direct sampling and analysis methods. This limitation is highly significant in the case of dogs due to their roaming and scavenging behavior, as illustrated by a case of lethal poisoning in a dog following exposure to microcystins at a lake shore site where a water sample collected within 24 hours of the exposure indicated low risk [12]. Recreational lake users such as swimmers, skiers and anglers who enter the water, livestock, and wildlife may also be at risk from localized hazards when other areas of a lake that were sampled may be safe, depending on the specific location where contact with the lake water occurs and the positioning of floating algal scums at the time. Knowledge of the specific locations of cyanobacterial biomass accumulations at the time when water contact occur are therefore critical to effective local risk management, and sUAS are likely to provide the most efficient means for generating the required data.

Two types of aircraft were used in these studies because the optimal aircraft type depends on the area that needs to be covered. Multirotor aircraft are typically limited to 25 minutes or less of flight duration with currently available battery technology. They also fly relatively slowly and are therefore limited in coverage per flight to 10s of acres. The ability to hover in place at low altitude can, however, be an advantage when specific sites need to be investigated in extremely high detail, or when suitable open areas for fixed wing aircraft takeoff and landing are not available. Fixed wing aircraft, on the other hand, are relatively efficient, fly longer and faster, and can cover larger areas of 100s of acres. Flying at an altitude of 400 feet, a fixed wing sUAS can typically cover about 600 acres within 25 to 40 minutes with a spatial resolution in the resulting images of less than 5 cm, while maintaining visual line of sight with the operator. Operations beyond visual line of sight require additional measures to ensure safe operations, and were outside the scope of this study. The costs of practical sUAS aerial photography platforms, and the complexity of their operation, have reduced dramatically in recent years. System cost and operational complexity are no longer insurmountable barriers to the use of sUAS at a local level. Airspace use regulations can be a significant barrier to sUAS use in countries where sUAS-specific regulations have not been developed, or where their use have been banned. Regulations associated with sUAS operations are, however, in flux in many parts of the world, and the trend is towards the creation of regulations that allow for the safe operation of sUAS, particularly when such operations are conducted with light-weight sUAS in rural areas, away from airports, at low altitude, and within visual line of sight.

The interpretation of data derived from sUAS can be approached in a two-tiered manner, first as qualitative assessment of single images and orthomosaics, and followed by quantitative assessment of cyanobacterial biomass concentrations based on cyanobacterial biomass density maps derived from the correlation between BNDVI and BPCV. Usefully, locally relevant risk assessment that lead to practical risk management decision can, in many cases, be based solely on qualitative assessment, particularly when the most important determination is the spatial distribution of harmful algal blooms at the time of the flight. Flights were conducted over Lake Centralia to demonstrate the utility of sUAS-based remote sensing in qualitative scouting for the presence of water surface regions with high levels of reflectance in the NIR band, which may indicate the presence of algae, and for the tracking of HAB distribution over time (Figure 1). The results indicated that sUAS-based remote sensing could be effective in this application.

Quantitative assessments are useful for tracking the density of blooms over time. It can be of value in quantitative risk assessment, and has the potential to be used in risk management decisions guided by predetermined policies regarding exposure limits. BPCV has advantages for field use because it requires relatively inexpensive, portable equipment, and very little operator training is needed to produce robust results. Although correlations between BNDVI and BPCV were strong in all three investigations, the model parameters differed. Differences could be attributed to cyanobacteria type differences, bloom stage differences, water differences, and differences in atmospheric conditions. Quantitative accuracy is therefore only possible when model calibration is performed using water samples collected at known locations at the time of the sUAS flight. The advantage of using a calibrated model in conjunction with a BNDVI map is that cyanobacterial biomass estimates can be extrapolated to the whole imaged water surface area, with a high degree of spatial resolution limited only by the resolution of the BNDVI map. Flights conducted at a low altitude using sUAS allows for very high spatial resolutions of 5 cm or less, depending on the flight altitude. It should be noted, however, that BPCV is not directly equivalent to cell density estimates derived from standard microscopy methods, and further work is needed to establish the relationship between PBCV and other cell density estimates. BPCV also does not provide information on the species composition of blooms, and traditional microscopy is still required to identify the cyanobacterial types and species composition involved in blooms.

A limitation of remote sensing methods is that they do not quantify toxin concentrations. Toxin concentrations are an essential component of risk assessment, and the sUAS-based remote sensing approach described here is therefore not suitable for risk assessment in isolation. The best use of sUAS is, arguably, as a complimentary source of data to monitor identified HABs in conjunction with other relevant data, and will be particularly useful in situations where the distribution pattern and surface density of a HAB needs to be characterized and tracked with a high level of spatial and temporal precision and accuracy after the organisms involved in the bloom have been characterized, and their potential to produce toxins have been evaluated by conventional means involving microscopy and toxin analysis on water samples.

There are environmental limitations on the use of sUAS-based remote sensing that need to be considered in the decision to deploy the technology and in the interpretation of data. Ideal atmospheric conditions are clear skies, and low wind velocity. Although data can be collected under cloudy conditions, it typically introduces variability in solar irradiance that may introduce excessive variability into the results independent of algal density. Clouds do not, however, compromise the ability to qualitatively characterize algal scum distribution patterns on the water surface or along the shoreline. Wind can be an important factor because it introduces wave-action at the water surface that degrades the consistency of reflectance values. The specific wind velocity that causes wave formation is variable and depends on the size of the water body, the wind direction, and the presence of obstacles to air movement. The presence of waves is, therefore, best evaluated locally, at the time of the intended flight. Sun angle also plays an important role in the quality and consistency of reflectance values obtained from water surfaces. Mirror-like reflections from the sun, when the sun is positioned above the sensor, causes an area of high reflectance values that are unrelated to algal density to appear on images. Flights should therefore be conducted at times when the sun angle is such that mirror-like sun reflections are not visible in images. Mid-morning and mid-afternoon are, therefore, optimal times in the day for conducting flights.

Reflectance values are influenced by the angles between the sensor, the light source, and the surface because most natural surfaces are non-Lambertian, leading to bidirectional reflectance artifacts in images [22]. Low altitude sensors using wide angle lenses are therefore associated with variations in results unrelated to algal density because the observation angle changes significantly depending on how far from the image center a surface point is. To reduce the influence of bidirectional reflectance, reflectance values were averaged for each surface point, using multiple images taken from different locations at the same altitude over the area of interest. The creation of a virtual surface model from multiple images is an essential step in data processing to enable efficient reflectance value averaging.

Atmospheric conditions, sun angles, and sun irradiance levels influence surface reflectance values, and vary over time. Sensor sensitivity may also change over time. Furthermore, internal radiometric sensor calibration as required for stable sensor sensitivity over time [23] is not feasible in converted consumer-grade sensors. Quantitative comparisons of reflectance values determined at different times therefore require that the data be normalized to a common standard. Semi-lambertian invariant target panels were deployed around livestock ponds for this purpose. The targets were not utilized for radiometric calibration in the current study because livestock ponds were not assessed over multiple time points, but it will be needed for data normalization to a common standard if comparisons over time are needed in future studies. It should be noted, however, that invariant target panels do not provide a complete solution to data normalization across time and location because panels do not account for certain sources of variation in the reflectance values of algae in water. These potential sources of error include variations in the optical properties of water depending on light absorption by organic matter and backscattering from suspended particulates [14], as well as the effects of non-Lambertian reflectance characteristics of panels that are amplified due to the use of wide angle lenses on sensors that are deployed at low altitude. These considerations support our recommendation that the sUAS approach described here is best used to compliment and expand the utility of water samples obtained at the time when flights are conducted. Aerial images processed into averaged reflectance orthomosaics can then be used to derive accurate surface algal density estimates for the entire area covered by the orthomosaic, and reduces the uncertainty associated with interpolation between sparse data points.

## 3. Experimental Section

Due to the presence of chlorophyll-a, light absorption and reflection patterns in cyanobacteria generally follow the patterns associated with green plants. Light in the visible spectrum is absorbed, with a relatively high absorption rate in the blue and red regions of the visible spectrum, while near infrared (NIR) light is strongly reflected. Absorption of NIR light by water provides a contrast in the NIR band between cyanobacteria at the water surface, and surrounding clear water. The contrast between clear water and cyanobacteria in color-infrared imagery can therefore be used to visually distinguish between clear water and cyanobacteria with a high degree of sensitivity, and the ratio between reflected blue light and reflected NIR can be used to quantify algal density at the water surface by creating a parameter called the blue normalized difference vegetation index (BNDVI; Equation (5)):

(5) BNDVI = (NIR − blue)/(NIR + blue)

BNDVI was calculated from JPEG-format images captured by a modified digital camera (Canon Powershot S100 NDVI, LDP LLC, Carlstadt, NJ, USA). The filters on the camera sensor were modified to allow visible blue and visible green light between 400 nm and 580 nm, and the visible red edge to near infrared transition between 680 nm to 780 nm, to pass to the sensor, while blocking visible red light between 580 nm and 680 nm, and NIR light above 780 nm.

Two aircraft types, fixed wing and multirotor, were used. The fixed wing aircraft was a Zephyr sUAS. It is a flying wing with a 137 cm wingspan (RitewingRC, Phoenix, AZ, USA), controlled with an Ardupilot Mega 2.6 (3DRobotics, San Diego, CA, USA). Modifications from the standard configuration included strengthening of the leading edge of the wing to withstand landing in rough vegetation by applying laminating film of 0.254 mm thickness, and the installation of a custom camera holder constructed out of expanded polypropylene foam. The multirotor was a DJI F550 controlled with a NAZA V2 (DJI Innovations, Shenzhen, China), fitted with a camera gimbal (Gaui Crane II, TSH Gaui Hobby Corporation, New Taipei City, Taiwan), and a real-time video system (ReadymadeRC LLC, Lewis Center, OH, USA).

Flight planning for fixed wing operations was done in Mission Planner, free software under the terms of the General Public License (Free Software Foundation, Boston, MA, USA). Flights were conducted at an altitude of 122 m, with flight line intervals of 33 m, a ground speed of 15 m/s, and an image interval of 3 s, to achieve front and side overlaps of 75%. Multirotor flights were flown manually, at 25–50 m altitude, to achieve image overlap of at least 75%. Four invariant target panels of 0.37 m^2^ were deployed around livestock ponds. The targets were made from particle board covered with matt acrylic latex paint (Behr Premium Plus #S-H-390, The Home Depot, Inc., Brandon, FL, USA). The targets provided semi-Lambertian surfaces with reflectance characteristics that fall within the typical reflectance value ranges of green vegetation for the bands detected by the modified digital camera used in the study.

Images were processed into averaged orthomosaics using Agisoft Photoscan Professional Version 1.0.4 build 1847 (Agisoft LLC, St. Petersburg, Russia). The image processing procedure involved the following steps: photo alignment using high accuracy and generic pair preselection, buiding a surface mesh using a high polygon count, and exporting an orthophoto in JPEG format using the average blending mode.

BNDVI maps were derived in ArcGIS Desktop 10.2.2.3552 (Esri Inc., Redlands, CA, USA), using the Raster Calculator tool in the Spatial Analyst extension. The procedure included importing NIR and blue data as separate raster layers, converting pixel values to floating-point values, and applying the BNDVI algorithm discussed above.

Surface water samples were scooped from the lake or pond water surface at the shoreline using a cup attached to a 1.8 m long handle. Locations were marked using white vinyl strips 6.5 cm wide and 90 cm long. Water samples were taken at consistent distances from the markers to facilitate identification of the sample locations on aerial images. Water samples were transferred to 50 mL polypropylene tubes (Environmental Express, Charlston, SC, USA) for storage in a cooled container, and analyzed within 48 h.

Cyanobacteria were identified to genus level based on microscopic morphology light microscopy (Olympus SZX16, Olympus Corporation, Center Valley, NJ, USA). Due to the need for a rapid quantitation method for surface cyanobacterial biomass that can be deployed under field conditions, a quantitation method was developed based on the buoyant portion of cells after using microscopy to confirm that the buoyant cells were cyanobacteria. Water samples were mixed by rapid inversion of the sample vial by hand at least 10 times, followed by immersion of one end of 75 mm capillary micro-hematocrit tubes (Cat. No. 21112, Sherwood Medical Industries, St. Louis, MO, USA) to draw water into the tubes to about 80% of the tube length, followed by sealing with clay (Seal-ease, Clay-Adams Inc., New York, NY, USA). Tubes were centrifuged for 10 minutes (International Centrifuge Model MB, International Equipment Company, Boston, MA, USA). BPCV was read as a percentage of the total sample volume using a micro-hematocrit tube reader (Critocap, Biological Research Inc., St. Louis, MO, USA). Fractions of a percent were derived from images produced using a stereo microscope (Olympus SZX16, Olympus Corporation, Center Valley, NJ, USA), and quantified by comparing the lengths of transects through the cell layer portion of the image and a 1% reference in the background.

To establish the correlation between BPCV and blue NDVI, a fresh sample of *Microcystis* algal scum derived from Centralia Lake, KS, with a BPCV of 50%, was serially diluted with tap water to 25%, 12.5%, 6.3% and 3.1%, and placed into a 96 well microplate (Cooke Microtiter, Cooke Engineering Company, Alexandria, VA, USA). Broad-spectrum light was produced with a halogen lamp source (Cole-Palmer Illuminator 41720, Cole-Palmer, Vernon Hills, IL, USA). The samples were imaged using a converted camera, followed by calculation of a blue NDVI for each sample.

## 4. Conclusions

sUAS-based remote sensing methods provide valuable information that is complimentary to HAB risk assessment data derived from traditional methods, and could improve risk management at the local level. It is particularly useful in situations where the distribution pattern and surface density of a HAB needs to be characterized and tracked with a high level of spatial and temporal precision and accuracy.

## References

[1] Paerl H.W., Fulton R.S., Moisander P.H., Dyble J. (2001). Harmful freshwater algal blooms, with an emphasis on cyanobacteria. Sci. World..

[2] De Figueiredo D.R., Azeiteiro U.M., Esteves S.M., Gonsalves F.J.M., Pereira M.J. (2004). Microcystin-producing blooms—A serious global public health issue. Ecotoxicol. Environ. Saf..

[3] Hudnell H.K. (2010). The state of US freshwater harmful algal blooms assessments, policy and legislation. Toxicon.

[4] Briand J.F., Jacquet S., Bernard C., Humbert J.F. (2003). Health hazards for terrestrial vertebrates from toxic cyanobacteria in surface water ecosystems. Vet. Res..

[5] Trevino-Garrison I., DeMent J., Ahmed F.S., Haines-Lieber P., Langer T., Ménager H., Ménager H., Neff J., van der Merwe D., Carney E. (2015). Human illnesses and animal deaths associated with freshwater harmful algal blooms—Kansas. Toxins.

[6] Dodds W.K., Bouska W., Eitzmann J.L., Pilger T.J., Pitts K.L., Riley A.J., Schloesser J.T., Thornbrugh D.J. (2009). Eutrophication of US freshwaters: Analysis of potential economic damages. Environ. Sci. Technol..

[7] Downing J.A., Watson S.B., McCauley E. (2001). Predicting Cyanobacteria dominance in lakes. Can. J. Fish. Aquat. Sci..

[8] Graham J.L., Jones J.R., Jones S.B., Downing J.A., Clevenger T.E. (2004). Environmental factors influencing microcystin distribution and concentration in the Midwestern United States. Water Res..

[9] Kanoshina I., Lips U., Leppanen J. (2003). The influence of weather conditions (temperature and wind) on cyanobacterial bloom development in the Gulf of Finland (Baltic Sea). Harmful Algae.

[10] Azevedo S.M.F.O., Carmichael W.W., Jochimsen E.M., Rinehart K.L., Lau S., Shaw G.R., Eaglesham G.K. (2002). Human intoxication by microcystins during renal dialysis treatment in Caruaru-Brazil. Toxicology.

[11] Chorus I., Bartram J. (1999). Toxic Cyanobacteria in Water: A Guide to Their Public Health Consequences, Monitoring and Management.

[12] Van der Merwe D., Sebbag L., Nietfeld J.C., Aubel M.T., Foss A., Carney E. (2012). Investigation of a *Microcystis aeruginosa* cyanobacterial freshwater harmful algal bloom associated with acute microcystin toxicosis in a dog. J. Vet. Diagn. Invest..

[13] Lunetta R.S., Schaeffer B.A., Stumpf R.P., Keith D., Jacobs S.A., Murphy M.S. (2014). Evaluation of cyanobacteria cell count detection derived from MERIS imagery across the eastern USA. Remote Sens. Environ..

[14] Gitelson A.A., Dall’Olmo G., Moses W., Rundquist D.C., Barrow T., Fisher T.R., Gurlin D., Holz J. (2008). A simple semi-analytical model for remote estimation of chlorophyll-*a* in turbid waters: Validation. Remote Sens. Environ..

[15] Moses W.J., Gitelson A.A., Perk R.L., Gurlin D., Rundquist D.C., Leavitt B.C., Barrow T.M. (2012). Estimation of chlorophyll-*a* concentration in turbid productive waters using airborne hyperspectral data. Water Res..

[16] Han L., Rundquist D.C. (1997). Comparison of NIR/RED ratio and first derivative of reflectance in estimating algal-chlorophyll concentration: A case study in a turbid reservoir. Remote Sens. Environ..

[17] Rundquist D.C., Han L., Schalles J.F., Peake J.S. (1996). Remote measurement of algal chlorophyll in surface waters: The case for the first derivative of reflectance near 690 nm. Photogramm. Eng. Remote Sens..

[18] Hunter P.D., Tyler A.N., Carvalho L., Codd G.A., Maberly S.C. (2010). Hyperspectral remote sensing of cyanobacterial pigments as indicators for cell populations and toxins in eutrophic lakes. Remote Sens. Environ..

[19] Keith D.J., Milstead B., Walker H., Snook H., Szykman J., Wusk M., Kagey L., Howell C., Mellanson C., Drueke C. (2012). Trophic status, ecological condition, and cyanobacteria risk of New England lakes and ponds based on aircraft remote sensing. J. Appl. Remote Sens..

[20] Wynne T.T., Stumpf R.P., Briggs T.O. (2013). Comparing MODIS and MERIS spectral shapes for cyanobacterial bloom detection. Int. J. Remote Sens..

[21] Gitelson A.A., Schalles J.F., Rundquist D.C., Schiebe F.R., Yacobi Y.Z. (1999). Comparative reflectance properties of algal cultures with manipulated densities. J. Appl. Phycol..

[22] Walthall C., Norman J., Welles J., Campbell G., Blad B. (1985). Simple equation to approximate the bidirectional reflectance from vegetative canopies and bare soil surfaces. Appl. Opt..

[23] Vogelmann J.E., Helder D., Morfitt R., Choate M.J., Merchant J.W., Bulley H. (2001). Effects of Landsat 5 Thematic Mapper and Landsat 7 Enhanced Thematic Mapper Plus radiometric and geometric calibrations and corrections on landscape characterization. Remote Sens. Environ..

